# The usefulness of the total metabolic tumor volume for predicting the postoperative recurrence of thoracic esophageal squamous cell carcinoma

**DOI:** 10.1186/s12885-022-10281-4

**Published:** 2022-11-15

**Authors:** Hayato Kaida, Takushi Yasuda, Osamu Shiraishi, Hiroaki Kato, Yutaka Kimura, Kohei Hanaoka, Minoru Yamada, Yuko Matsukubo, Masakatsu Tsurusaki, Kazuhiro Kitajima, Satoshi Hattori, Kazunari Ishii

**Affiliations:** 1 Department of Radiology, Kindai University Faculty of Medicine, 377-2 Ohnohigashi, Osaka-Sayama, Osaka, 589-8511 Japan; 2 Division of Positron Emission Tomography, Institute of Advanced Clinical Medicine, Kindai University Hospital, Osaka-Sayama, Osaka, 589-8511 Japan; 3 Department of Surgery, Kindai University Faculty of Medicine, Osaka-Sayama, Osaka, 589-8511 Japan; 4 Department of Radiology, Division of Nuclear Medicine and PET Center, Hyogo College of Medicine, Nishinomiya, Hyogo 663-8501 Japan; 5 Department of Biomedical Statistics, Osaka University Graduate School of Medicine, Suita, Japan; 6 Institute for Open and Transdisciplinary Research Initiative, Osaka University, Suita, Osaka, 565-0871 Japan

**Keywords:** Esophageal squamous cell carcinoma, ^18^F-FDG-PET/CT, TMTV

## Abstract

**Background:**

Induction or adjuvant therapies are not always beneficial for thoracic esophageal squamous cell carcinoma (ESCC) patients, and it is thus important to identify patients at high risk for postoperative ESCC recurrence. We investigated the usefulness of the total metabolic tumor volume (TMTV) for predicting the postoperative recurrence of thoracic ESCC.

**Methods:**

We retrospectively analyzed the cases of 163 thoracic ESCC patients (135 men, 28 women; median age of 66 [range 34–82] years) treated at our hospital in 2007–2012. The TMTV was calculated from the fluorine-18 fluorodeoxyglucose (^18^F-FDG) uptake in the primary lesion and lymph node metastases. The optimal cut-off values for relapse and non-relapse were obtained by the time-dependent receiver operating curve analyses. Relapse-free survival (RFS) was evaluated by the Kaplan-Meier method, and between-subgroup differences in survival were analyzed by log-rank test. The prognostic significance of metabolic parameters and clinicopathological variables was assessed by a Cox proportional hazard regression analysis. The difference in the failure patterns after surgical resection was evaluated using the χ^2^-test.

**Results:**

The optimal cut-off value of TMTV for discriminating relapse from non-relapse was 3.82. The patients with a TMTV ≥3.82 showed significantly worse prognoses than those with low values (*p* < 0.001). The TMTV was significantly related to RFS (model 1 for preoperative risk factors: TMTV: hazard ratio [HR] =2.574, *p =* 0.004; model 2 for preoperative and postoperative risk factors: HR = 1.989, *p =* 0.044). The combination of the TMTV and cN0–1 or pN0–1 stage significantly stratified the patients into low-and high-risk recurrence groups (TMTV cN0–1, *p* < 0.001; TMTV pN0–1, *p =* 0.004). The rates of hematogenous and regional lymph node metastasis were significantly higher in the patients with TMTV ≥3.82 than those with low values (hematogenous metastasis, *p* < 0.001, regional lymph node metastasis, *p =* 0.011).

**Conclusions:**

The TMTV was a more significantly independent prognostic factor for RFS than any other PET parameter in patients with resectable thoracic ESCC. The TMTV may be useful for the identifying thoracic ESCC patients at high risk for postoperative recurrence and for deciding the patient management.

**Supplementary Information:**

The online version contains supplementary material available at 10.1186/s12885-022-10281-4.

## Background

Esophageal cancer ranks seventh in terms of incidence and sixth in mortality in the world, and it has shown high rates of recurrence and distant metastasis in patients with pathological lymph node metastases (LNMs) [1, 2]. In western countries, neoadjuvant chemoradiotherapy (NACRT) plus surgery is considered the standard treatment for patients with resectable locally advanced esophageal cancer [3]. In Japan, neoadjuvant chemotherapy (NAC) is another standard therapy for patients with stage II/III esophageal squamous cell carcinoma (ESCC) [4]. Unfortunately, these induction therapies are not always beneficial for all patients. It is therefore crucial to identify patients who are at high risk for recurrence, at the initial staging. The tumor-node metastasis (TNM) stage is currently the most valued factor for deciding patient management and predicting patient survival, but this system does not include biological information, and many patients even at the early stage of esophageal cancer show early recurrence and metastasis. A more selective biomarker for predicting ESCC recurrence and metastasis is needed.

The maximum standardized uptake value (SUVmax) is a commonly used semi-quantitative parameter used to evaluate malignant tumors on fluorine-18 fluorodeoxyglucose positron emission tomography/computed tomography (^18^F-FDG-PET/CT). Although this parameter reflects the highest pixels within a designated region of interest, it does not always reveal the glucose metabolism within the whole tumor. Total lesion glycolysis (TLG) and the metabolic tumor volume (MTV) are representative PET volumetric parameters. The MTV is an important parameter to determine the local tumor burden and the total tumor burden obtained by a commonly fixed threshold method, and it is usually expressed in milliliters or cubic centimeters [5]. The TLG value provides information about total tumor glycolysis calculated from the following formula: SUVmean × MTV [5]. In other words, the TLG takes into account the intensity of radiotracer uptake and a volumetric factor [5].

For patients with esophageal cancer, a change in the MTV of the primary tumor was recently reported to be clinically useful in predicting both long-term survival and histological response to NAC, and the MTV of the primary tumor has been suggested to be a significant prognostic factor in patients treated with chemoradiotherapy (CRT) [6, 7]. Thus, the relationship between the ^18^F-FDG uptake in the primary tumor and the patients’ survival has been investigated. However, the number of pathological LNMs is a crucial factor for postoperative survival, and the preoperative ^18^F-FDG uptake in lymph nodes status has been suggested to be significantly associated with the size and the number of pathological LNMs and survival in patients with resectable ESCC [8]. It is critically important to determine the correlation between prognosis and the ^18^F-FDG uptake in both the primary tumor and LNMs.

The measurement of the total metabolic tumor volume (TMTV, which gives an estimation of the total tumor burden) has gained attention, and the usefulness of the TMTV for predicting tumor responses and prognoses has been reported in several malignant tumors [9, 10]. The TMTV and whole-body TLG (wTLG) have been reported to be independent prognostic factors in esophageal cancer patients treated with CRT [11]. To our knowledge, there have been few investigations about the relationship between recurrence and TMTV in ESCC patients undergoing curative surgical resection. We conducted the present study to investigate the usefulness of the TMTV compared with other PET parameters (i.e., the SUVmax, lean body mass SUVpeak [SULpeak], and TLG) for predicting postoperative recurrence in thoracic ESCC patients.

## Patients and methods

### Patients

**Fig. 1:**
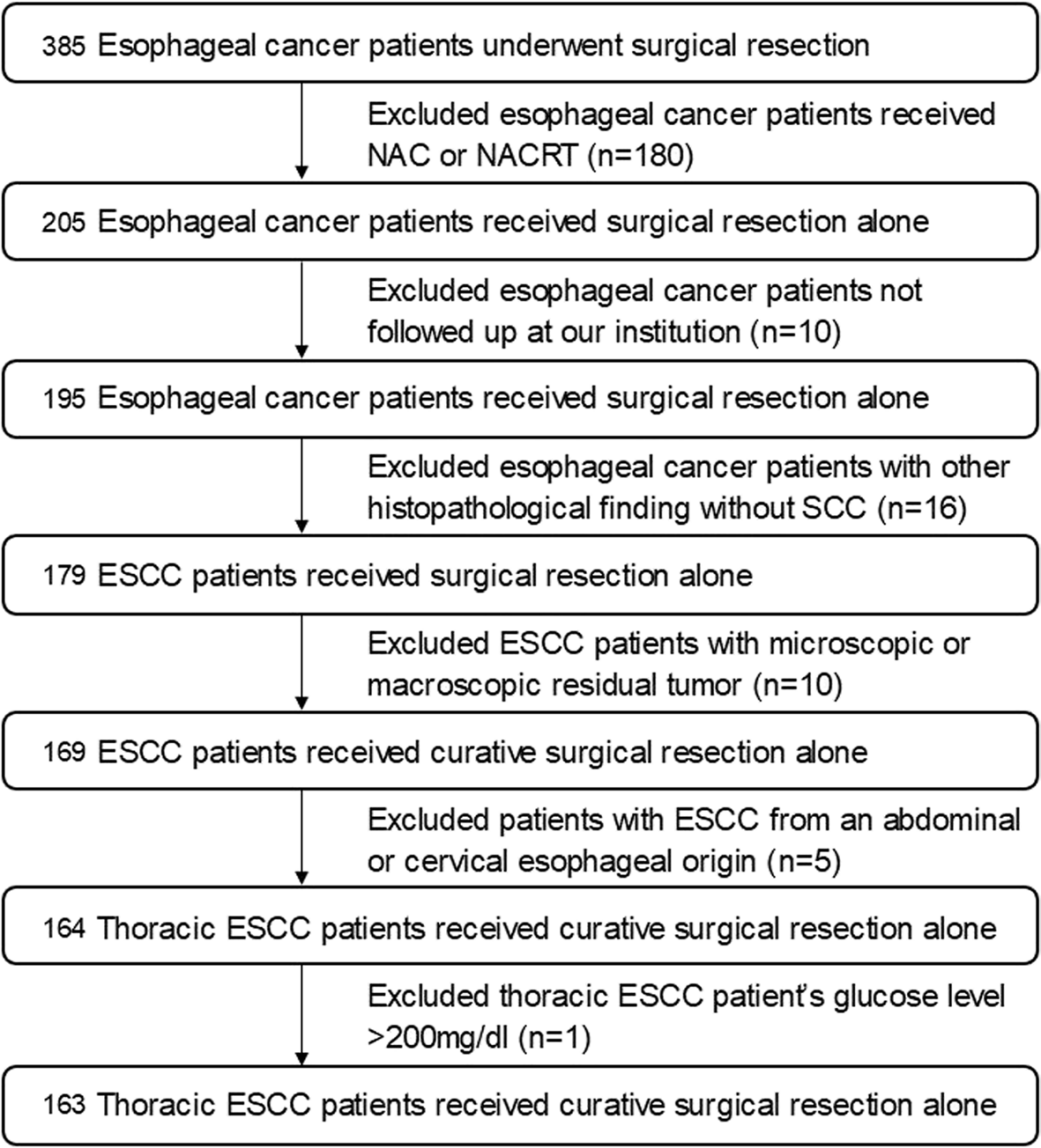
The flow chart demonstrates the patient selectin on this study. During the period from December 2007 to December 2012, 385 consecutive esophageal cancer patients underwent a surgical resection at our institution. The patients with other histopathological finding without SCC were excluded because SCC is the predominant histologic type of esophageal cancer, accounting for approx. 90% of all cases; non-SCC patients are rare. SCC: squamous cell carcinoma, NAC: neoadjuvant chemotherapy, NACRT: neoadjuvant chemoradiotherapy, ESCC: esophageal squamous cell carcinoma

The eligibility of patient for this study is all of the included thoracic ESCC patients who underwent a complete curative surgical resection without prior induction treatment during the period from December 2007 to December 2012. Our inclusion criteria are demonstrated in Fig. 1. On treatment strategy of thoracic ESCC patients in our institution, the patients with cStage I underwent curative surgical resection without NAC. In patients with cStage II,III, IV (M1LYM), NAC followed by radical surgical resection was considered as first line therapy, and whether these patients were candidate for NAC was determined considering ^18^F-FDG PET/CT result. The eligibility of NAC is following criteria; cT3 or less, resectable, and histologically confirmed thoracic ESCC, with presence of PET-N-positive (^18^F-FDG uptake on PET observed in lymph nodes within a three-field region, including M1LYM of the supraclavicular, cervical paratracheal and celiac artery lymph nodes), no evidence distant metastasis, age 20–79 years, Eastern Cooperative Oncology Group performance status 0–1, adequate hematological and visceral function, no severe diabetes mellitus, no severe mental disorder, no previous chemo-or radiotherapy for any malignancies, and no active malignant disease [12]. In case of PET-N-negative patients, curative surgical resection without NAC was done. Unfortunately, provided that intramural metastasis was present, or lymph node metastasis was suspected from CT of the neck, chest and abdomen in spite of PET-N-negative, NAC was also conducted to thoracic ESCC patients who met these conditions. The final study population was 163 patients (135 men, 28 women) with the median age of 66 (range 34–82) years. The TNM stages were established using the seventh edition of the UICC for staging esophageal cancer [13]. The study protocol was conducted according to the Declaration of Helsinki and was approved by the Ethics Committee, Kindai University Faculty of Medicine (approval no. 28–005), which waived the need for written informed consent due to the study’s retrospective design.

### ^18^F-FDG-PET/CT imaging acquisition

An integrated PET/CT scanner (Biograph/Somatom Emotion Duo, Siemens Medical Solutions, Hoffmann Estates, IL, USA) was used for the data acquisition. All PET images were acquired using a matrix of 128 × 128 pixels. The time needed for one bed position (162 mm in the z-direction) scan was 120–150 sec. The voxel dimensions were 3.9 mm × 3.9 mm × 5.0 mm.

The CT data were used for attenuation correction and lesion localization. After both the CT and emission images were transmitted, the images were reconstructed using the standard normal reconstruction protocol based on the ordered subset expectation maximization method and a 5-mm Gaussian filter. Before receiving the ^18^F-FDG injection, the patient fasted for 4 hr. We included six patients with diabetes mellitus because hyperglycemia with a pre-scan blood glucose level at 110–200 mg/dL is not associated with significantly different SUVmax and SUVmean values in the tumor [14]. None of the patients had a glucose level > 200 mg/dL. All patients were administered a median dose of 156.3 MBq (adjusted for body weight, range 87–243 MBq) via the antecubital vein, and they were then instructed to rest quietly for approx. 60 min. The median duration between the surgical resection and ^18^F-FDG-PET/CT was 23 days (range 1–56 days).

### ^18^F-FDG-PET/CT image analysis

^18^F-FDG-PET/CT images were displayed on an Advantage Windows Workstation (GE Healthcare, Milwaukee, WI) on which volumes of interest (VOIs) were semi-automatically drawn over the entire abnormal uptake of the primary tumor and any lymph node metastasis on axial images. The SUVmax and the SUVmean were defined as the maximum and mean activity, respectively, of the concentration of ^18^F-FDG uptake in the primary tumor or lymph node metastasis. We used 40% as the threshold of the maximum peak activity within the lesions to delineate the MTV [15].

The SUVpeak was calculated in a 1.2-cm-dia. Region of interest (ROI) placed on the uptake lesions and defined as the average SUV inside an ROI centered in the highest uptake volume of the lesion [5]. The SUVpeak was normalized to the SULpeak as follows: SUVpeak × [lean body mass] / [total body mass]. Lean body mass was calculated based on a published formula [16]. The border of the VOI was adjusted manually by avoiding overlap with the adjacent ^18^F-FDG-avid structures, physiological uptake, and lesions. The SUVmax, MTV, TLG, and SULpeak of each lesion were automatically calculated and recorded on the workstation.

For the evaluations of LNMs, we considered paraesophageal or other mediastinal and abdominal lymph nodes with ^18^F-FDG uptake that was higher than the mediastinal blood pool activity on visual inspection as metastasis, and we assessed PET-N-positive and PET-N-negative based on ^18^F-FDG PET/CT finding and the respective patient’s postoperative histopathological findings. The highest SUVmax (peakSUVmax) and the highest SULpeak (hSULpeak) were selected from the ^18^F-FDG uptake in the primary tumor and LNMs, respectively. The TMTV and the wTLG were calculated by summing the TLG (or MTV) of the primary tumor and the TLG (or MTV) of all LNMs (Suppl. Fig.S1).

### Surgical treatment and follow-up

All patients underwent a transthoracic subtotal esophagostomy, and an esophageal reconstruction was done using a gastric tube (149 patients) or a pedicle jejunum (14 patients due to simultaneous total gastrectomy or history of distal gastrectomy). A three-field lymphadenectomy was performed for patients with supraclavicular or recurrent laryngeal nerve LNMs or with a primary tumor located in the upper-third thoracic esophagus. The surviving patients were followed up at our outpatient department every 3–6 months for the first 5 years, and then on an annual basis. CT scans of the neck, chest, and abdomen were conducted 2×/year, and endoscopy was performed 1×/year.

### Statistical analyses

Relapse-free survival (RFS) was defined as the length of time from the date of the patient’s surgery until the date of the first evidence of relapse or the date of death from any cause. As the event occurrence is time-dependent, we performed a time-dependent receiver operating characteristics curve (ROC) analysis for censored survival data and relapse and used the area under the ROC curve (AUC) as the criterion [17, 18]. A larger AUC indicates better predictability of the time to an event. An AUC of 0.5 indicates no predictive ability, and a value of 1 represents perfect ability [17]. The optimal cut-off values for PET/CT parameters for the patients’ RFS were determined by a time-dependent ROC analysis. We used the χ^2^-test to evaluate the differences in the noncontinuous data between all metabolic parameters and various clinicopathological variables or failure patterns after surgical resection.

We used univariate and multivariable Cox proportional hazard models to evaluate the effects of metabolic parameters on the patients’ RFS. However, due to the high collinearity among all PET parameters (Spearman correlation test; *r* = 0.82–0.99), we performed two models of multivariate analysis using the PET parameters one-by-one. Model 1 consisted of preoperative risk factors such as age, gender, location, and clinical TNM stage. Model 2 was composed of both preoperative risk factors and the pathological TNM stage. Significant univariate variables (*p <* 0.05) were included in the multivariate analysis.

Using the cut-off values, we estimated the survival functions of RFS for the low-and high-risk groups by the Kaplan-Meier method, and we compared these functions by the log-rank test. Differences at *p* < 0.05 were regarded as significant. Statistical analyses were conducted using SPSS Statistics 27 software (IBM, Armonk, NY), and R v3.4.1; downloadable at https://cran.r-roject.org/bin/windows/base/old/3.4.1/.

## Results

### Time-dependent ROC analysis for RFS

**Fig. 2:**
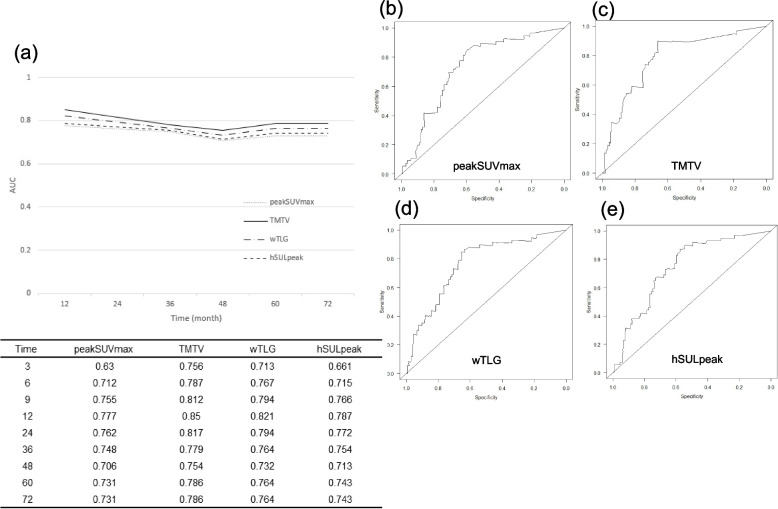
**a** The results of the time-dependent ROC curve analysis for the prediction of RFS regarding all PET parameters **b-e**. The time-dependent ROC curve analysis for the determination of cut-off values of four PET parameters for predicting 72 months RFS. AUC: the area under the curve, hSULpeak: highest lean body mass peak standardized uptake value, peakSUVmax: peak maximum standardized uptake value, PET: positron emission tomography, RFS: relapse-free survival, ROC: receiver operating characteristics curve, TMTV: total metabolic tumor volume, wTLG: whole-body total lesion glycolysis

We conducted a time-dependent ROC analysis to compare the PET parameters for predicting recurrence, and the results provided the AUC for each follow-up time. The resultant AUC data for the patients’ RFS are depicted in Fig. 2. The best accuracy for predicting recurrence until 72 months was obtained with TMTV (AUC: 0.786–0.85), wTLG (AUC: 0.732–0.821), hSULpeak (AUC: 0.713–0.787), and peakSUVmax (AUC: 0.706–0.777). The optimal cut-off values for relapse and non-relapse until 72 months were as follows. PeakSUVmax: 4.43, hSULpeak: 2.64, TMTV: 3.82, wTLG: 13.46.

### Patient characteristics and metabolic parameters

The patients’ characteristics and clinicopathological variables are summarized in Table 1. Sixty-three of the 163 patients did not receive NAC despite being at cStage II, III, or IV, and twenty-six of the 63 patients who were PET-N-negative did not undergo NAC because PET-N-negative ESCC patients have been reported to be at significantly lower risk for postoperative recurrence and to have a higher survival rate without NAC [8]. The PET-N-positive patients with resectable tumors clinically considered candidates for NAC, and this therapy was performed to these patients because they are likely to have higher rate of postoperative recurrence and distant metastasis, and NAC significantly suppressed postoperative recurrence in thoracic ESCC patients [8, 12]. Twelve patients with PET-N-negative and twenty-five patients with PET-N-positive were considered as candidate for NAC, unfortunately this therapy was not performed based on several medical reasons (Table S1). Regarding PET-N-positive, seven patients underwent curative surgical resection without NAC as local disease because of lymph node metastasis adjacent to primary tumor.

**Table 1:** The characteristic of the thoracic ESCC patients (*n =* 163)

Age, yrs. (median)	66 (range 34–82)
Gender, males/females	135 / 28
Location: upper/middle/lower	25 / 97 / 41
Clinical stage:	
cT1/cT2/cT3	87 / 40 / 36
cN0/cN1/cN2	113 / 42 / 8
cM0/cM1(LYM)	162 / 1
cStage (I/II/III/IV)	100 / 35 / 27 / 1
Pathological stage:	
pT1 / pT2 / pT3 / pT4	93 / 21 / 47 / 2
pN0 / pN1 / pN2 / pN3	88 / 45 / 22 / 8
pM0 / pM1(LYM)	159 / 4
pStage, I / II / III / IV	75 / 41 / 37 / 10
Postoperative therapy:	
Absent/present	127 / 36
Chemotherapy	24
Chemotherapy and cancer-specific vaccine therapy	5
Cancer-specific vaccine therapy	5
CRT	2
Median follow-up duration, months (range)	60.0 (3–72)
Recurrence, negative/positive	112 / 51
Censored patients/death	104 / 59

*CRT* Chemoradiotherapy, *LYM* Supraclavicular lymph node metastasis, *ESCC* Esophageal squamous cell carcinoma

The clinical and pathological advanced stages (except for the cM stage or pM stage), the number of ^18^F-FDG-positive lymph node metastases, and the rate of recurrence were significantly different between the high cut-off value groups and the low cut-off value groups for all four metabolic parameters (*p* < 0.001 for all). Patient age, gender, and tumor location were not significantly associated with any of the metabolic parameters. These data are shown in Supplementary Tables S2 and S3.

### The relationships between the PET parameters and survival

The median follow-up period of all patients was 60 months (range 3–72 months). Fifty-one of the 163 patients (31.1%) developed recurrence, and 60 (36.6%) patients died. The 5-year RFS rate for this patient population was 57.9%. The results of the log-rank tests of the Kaplan-Meier survival curves for 5-year RFS regarding all PET parameters and clinicopathological variables are shown in Table 2. Regarding the 5-year RFS, the survival of the patients with all low values on the four metabolic parameters of peakSUVmax, hSULpeak, TMTV, and wTLG were significantly longer than those of the high-value groups (*p* < 0.001 for all). There were also significant differences in the RFS in relation to the cTstage, cN stage, and pTNM stage.

**Table 2:** Log-rank test of PET parameters and clinicopathological factors for relapse-free survival (RFS)

Parameters	n	5-yrsurvival rate	***p***-value
Age:			
< 66	78	59.9%	0.514
≥ 66	85	55.6%	
Gender:			
Male	135	58.8%	0.373
Female	28	51.8%	
Location:			
Ut-Mt	121	58.0%	0.878
Lt	42	56.6%	
Mt-Lt	138	59.6%	0.134
Ut	25	47.7%	
cT stage:			
cT1–2	128	66.0%	< 0.001
cT3	35	27.1%	
cN stage:			
cN0	113	68.3%	< 0.001
cN1–2	50	32.8%	
cM stage:			
cM0	162	58.0%	0.165
cM1(LYM)	1	NA	
pT stage:			
pT1–2	114	69.8%	< 0.001
pT3–4	49	28.2%	
pN stage:			
pN0–1	133	67.4%	< 0.001
pN2–3	30	14.0%	
pM stage:			
pM0	159	58.5%	0.035
pM1(LYM)	4	NA	
peakSUVmax:			
< 4.43	71	74.9%	< 0.001
≥ 4.43	92	44.1%	
TMTV:			
< 3.82	76	77.9%	< 0.001
≥ 3.82	87	39.7%	
wTLG:			
< 13.46	78	73.1%	< 0.001
≥ 13.46	85	43.2%	
hSULpeak:			
< 2.64	68	75.2%	< 0.001
≥ 2.64	95	44.8%	

*hSULpeak* Highest lean body mass peak standardized uptake value, *Lt* Lower thoracic esophagus, *Mt* Middle thoracic esophagus, *LYM* Supraclavicular lymph node metastasis, *PeakSUVmax* Peak maximum standardized uptake value, *PET* Positron emission tomography, *TMTV* Total metabolic tumor volume, *Ut* Upper thoracic esophagus, *wTLG* Whole-body total lesion glycolysis

The results of the univariate Cox proportional hazard models for RFS are provided in Table 3. The univariate analysis showed significant correlations between RFS and the cTstage, cN stage, peakSUVmax, TMTV, wTLG, hSULpeak, pT stage, pN stage, and pM stage. In the multivariable analysis for both models, TMTV remained significantly correlated with RFS (model 1: hazard ratio [HR] = 2.574, 95% confidential interval [95%CI] 1.338–4.826, *p =* 0.004; model 2: HR = 1.989, 95%CI 1.018–3.885, *p =* 0.044). Unfortunately, other PET parameters were not significantly associated with RFS for both models (Tables 4 and 5).

**Table 3:** Univariate Cox regression analysis for relapse-free survival (RFS)

	HR (95%CI)	***p***-value
Gender, male/female	0.761 (0.416–1.393)	0.377
Age, < 66 / ≥66	1.171 (0.726–1.887)	0.517
Tumor location:		
UtMt / Lt	1.076 (0.628–1.845)	0.789
Ut / MtLt	0.648 (0.360–1.167)	0.148
Clinical stage:		
cT stage, T1–2 / T3	3.543 (2.151–5.838)	< 0.001
cN stage, N0 / N1–2	0.301 (0.185–0.488)	< 0.001
cM stage, M0 / M1(LYM)	0.274 (0.038–1.998)	0.202
PET parameters:		
peakSUVmax, < 4.43 / ≥4.43	3.346 (1.929–5.805)	< 0.001
hSULpeak, < 2.64 / ≥2.64	3.325 (1.896–5.834)	< 0.001
TMTV, < 3.82 / ≥3.82	4.019 (2.313–6.982)	< 0.001
wTLG, < 13.46 / ≥13.46	2.996 (1.786–5.024)	< 0.001
Pathological stage:		
pT stage, T1–2 / T3–4	0.262 (0.162–0.423)	< 0.001
pN stage, N0–1 / N2–3	0.211 (0.127–0.350)	< 0.001
pM stage, M0 / M1(LYM)	0.308 (0.096–0.987)	0.048

Abbreviations are explained in the earlier table footnotes

**Table 4:** Model 1 for the multivariate Cox regression analysis for RFS

	peakSUVmax HR (95%CI)	***p***-value	hSULpeak HR (95%CI)	***p***-value	TMTV HR (95%CI)	***p***-value	wTLG HR (95%CI)	***p***-value
cT stage, T1–2 / T3	1.980 (1.126–3.480)	0.018	1.995 (1.137–3.499)	0.016	1.733 (0.987–3.043)	0.056	2.081 (1.167–3.771)	0.013
cN stage, N0 / N1–2	0.522 (0.297–0.914)	0.023	0.515 (0.295–0.900)	0.020	0.544 (0.316–0.939)	0.029	0.498 (0.277–0.897)	0.020
cM stage, M0 / M1(LYM)								
peakSUVmax, < 4.43 / ≥4.43	1.907 (0.984–3.699)	0.056						
hSULpeak, < 2.64 / ≥2.64			1.916 (0.987–3.719)	0.055				
TMTV, < 3.82 / ≥3.82					2.574 (1.338–4.826)	0.004		
wTLG, < 13.46 / ≥13.46							1.560 (0.802–3.037)	0.19

**Table 5:** Model 2 for the multivariate Cox regression analysis for RFS

	peakSUVmax HR (95%CI)	***p***-value	hSULpeak HR (95%CI)	***p***-value	TMTV HR (95%CI)	***p***-value	wTLG HR (95%CI)	***p***-value
cT stage, T1–2 / T3	1.355 (0.688–2.670)	0.337	1.354 (0.687–2.667)	0.381	1.214 (0.616–2.392)	0.530	1.377 (0.693–2.737)	0.362
cN stage, N0 / N1–2	0.762 (0.410–1.414)	0.762	0.764 (0.413–1.411)	0.390	0.765 (0.426–1.375)	0.371	0.696 (0.368–1.318)	0.266
cM stage, M0 / M1(LYM)								
peakSUVmax, < 4.43 / ≥4.43	1.501 (0.718–3.139)	0.280						
hSULpeak, < 2.64 / ≥2.64			1.543 (0.743–3.203)	0.245				
TMTV, < 3.82 / ≥3.82					1.989 (1.018–3.885)	0.044		
wTLG, < 13.46 / ≥13.46							1.115 (0.518–2.400)	0.781
pT stage, T1–2 / T3–4	0.535 (0.263–1.088)	0.084	0.537 (0.266–1.084)	0.083	0.524 (0.271–1.013)	0.055	0.477 (0.225–1.012)	0.054
pN stage, N0–1 / N2–3	0.352 (0.195–0.636)	0.001	0.351 (0.193–0.630)	0.001	0.401 (0.220–0.730)	0.003	0.356 (0.198–0.640)	0.001
pM stage, M0 / M1(LYM)	0.637 (0.194–2.091)	0.457	0.638 (0.194–2.094)	0.458	0.542 (0.165–1.781)	0.313	0.604 (0.184–1.980)	0.405

Abbreviations are explained in the earlier table footnotes

**Fig. 3:**
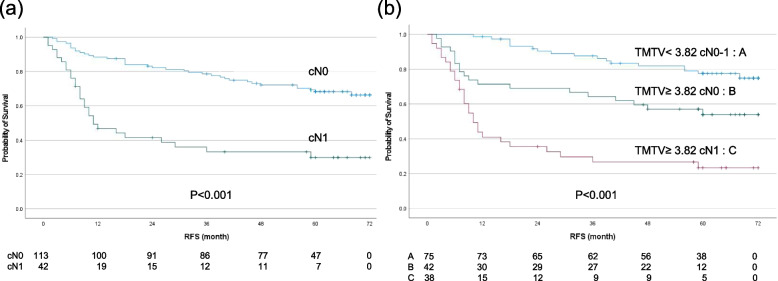
Kaplan-Meier estimates of survival functions for RFS in the pN0–1 group (**a**) and the combination of TMTV < 3.8 cN0–1, TMTV ≥3.8 cN0, and TMTV ≥3.8 cN1 groups in 155 thoracic ESCC patients (**b**). *P*-values were determined by the log-rank test

Based on the result of the multivariate analysis, we investigated the Kaplan-Meier survival curves for the RFS related to the combination of the TMTV and cN stage or pN stage. The 5-year RFS rate of the 155 patients with cN0 and cN1 disease was 58.2%; the 5-year RFS rate of the patients with cN0 was 68.3%, and that of the patients with cN1 was 30.0% (*p* < 0.001). The combination of TMTV and cN0–1 stage was significantly associated with RFS (*p* < 0.001). For TMTV and cN stage, the 5-year RFS rate of the patients with TMTV < 3.82 cN0–1, TMTV ≥3.82 cN0, and TMTV ≥3.82 cN1 were 77.6, 53.9, and 23.3%, respectively (Fig. 3). The characteristics of these subgroups are shown in Table 6.

**Table 6:** The characteristics of the thoracic ESCC patients with combined TMTV and cN0–1

	TMTV < 3.82 cN0–1	TMTV ≥ 3.8 cN0	TMTV ≥ 3.8 cN1
Total patients	75	42	38
Age, < 66 / ≥66	37 / 38	22 / 20	15 / 23
Gender, males/females	66 / 9	34 / 8	29 / 9
Location: upper/middle/lower	11 / 48 / 16	5 / 25 / 12	7 / 19 / 12
Clinical stage:			
cT1 / cT2 / cT3	67 / 7 / 1	15 / 15 / 12	4 / 15 / 19
cM0 / cM1(LYM)	75 / 0	42 / 0	38 / 0
cStage, I / II / III / IV	70 / 5 / 0 / 0	30 / 11 / 1 / 0	0 / 19 / 19 / 0
Recurrence, negative / positive	69 / 6	26 / 16	13 / 25

*ESCC* Esophageal squamous cell carcinoma, *TMTV* Total metabolic tumor volume, *LYM* Supraclavicular lymph node metastasis

The number of patients with cStage I in the TMTV < 3.82 cN0–1 group was higher than those of all of the other groups, and that of the patients with cStage II/III was higher in the TMTV ≥3.82 cN1group was higher than those of the other two groups. However, the TMTV ≥3.82 cN0 group had 30 patients (71.4%) with cStage I. Regarding these analyses, we did not separate the patients with TMTV < 3.8 into cN0 and cN1 because the number of patients with TMTV < 3.82 cN1 was only four patients who had no recurrent lesions or died within 72 months of follow-up.

**Fig. 4:**
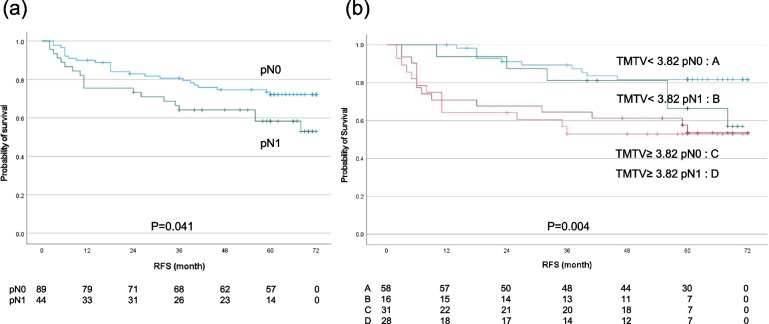
Kaplan-Meier estimates of survival functions for RFS in the pN0–1 group (**a**) and the combination of TMTV < 3.8 pN0, TMTV < 3.8 pN1, TMTV ≥3.8 pN0, and TMTV ≥3.8 pN1 groups in 133 thoracic ESCC patients (**b**). *P*-values were determined by the log-rank test

For the subgroup of 133 patients with pN0 and pN1 disease, the 5-year RFS rate of all patients was 67.4%, and the 5-year RFS rates of the patients with pN0 and those with pN1 were 72.2 and 57.3%, respectively (*p =* 0.041). The combination of the TMTV and pN0–1 stage significantly stratified high-risk patients by RFS (*p =* 0.004) in the following order: TMTV < 3.82 pN0 (5-yr RFS, 81.8%), TMTV < 3.82 pN1 (66.5%), TMTV ≥3.82 pN0 (53.6%), and TMTV ≥3.82 pN1 (52.9%) (Fig. 4). The characteristics of the TMTV and pN0–1 group are summarized in Table 7. The TMTV < 3.82 pN0 group included more thoracic ESCC patients with cStage I and pStage I than all of the other groups. The TMTV ≥3.82 pN0 group and the TMTV ≥3.82 pN1 group had more cStage II or III patients than the other two groups, and the TMTV ≥3.82 pN1 group had more pStage II–IV patients than all of the other groups.

**Table 7:** The characteristics of the thoracic ESCC patients with combined TMTV and pN0–1

	TMTV < 3.82 pN0	TMTV < 3.82 pN1	TMTV ≥ 3.8 pN0	TMTV ≥ 3.8 pN1
Total patients	58	16	31	28
Age, < 66 / ≥66	27 / 31	10 / 6	16 / 15	14 / 14
Gender, males / females	50 / 8	15 / 1	24 / 7	22 / 6
Location: upper / middle / lower	8 / 35 / 15	4 / 11 / 1	5 / 20 / 6	3 / 13 / 12
Clinical stage:				
cT1 / cT2 / cT3	53 / 5 / 0	12 / 3 / 1	10 / 13 / 8	7 / 7 / 14
cN0 / cN1 / cN2	55 / 2 / 1	14 / 2 / 0	25 / 6 / 0	14 / 10 / 4
cM0 / cM1(LYM)	58 / 0	16 / 0	31 / 0	28 / 0
cStage, I / II / III / IV	55 / 2 / 1 / 0	13 / 3 / 0 / 0	20 / 7 / 4 / 0	9 / 9 / 10 / 0
Pathological stage:				
pT1 / pT2 / pT3 / pT4	52 / 5 / 1 / 0	12 / 1 / 3 / 0	13 / 5 / 13 / 0	9 / 5 / 12 / 2
pM0 / pM1(LYM)	58 / 0	15 / 1	31 / 0	28 / 0
pStage, I / II / III / IV	57 / 1 / 0 / 0	0 / 13 / 3 / 0	18 / 13 / 0 / 0	0 / 14 / 12 / 2
Recurrence, negative / positive	54 / 4	14 / 2	21 / 10	15 / 13

*ESCC* Esophageal squamous cell carcinoma, *TMTV* Total metabolic tumor volume, *LYM* Supraclavicular lymph node metastasis

On the other hand, the TMTV ≥3.82 pN0 group had 20 patients (67.7%) with cStage I and 18 patients (58.1%) with pStage I, and the TMTV ≥3.82 pN1 group had nine patients (32.1%) with cStage I. The numbers of patients with pN0 and pN1 undergoing postoperative adjuvant chemotherapy were as follows; TMTV < 3.82 pN0: 0% (0/58), TMTV < 3.82 pN1: 35.3% (5/16), TMTV ≥3.82 pN0 0% (0/31), and TMTV ≥3.82 pN1: 46.4% (13/28).

**Fig. 5:**
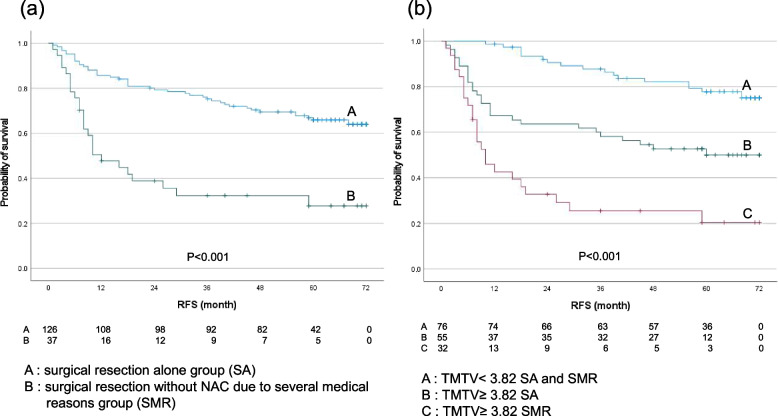
Kaplan-Meier estimates of survival functions for RFS in the group of SA and SMR (**a**) and the combination of TMTV < 3.8 SA and SMR, TMTV ≥3.8 SA, and TMTV ≥3.8 SMR groups in 163 thoracic ESCC patients (**b**). *P*-values were determined by the log-rank test. SA: surgical resection alone, SMR: surgical resection without NAC due to several medical reasons

In addition, we investigated the relationship between the patient group about surgical resection alone (SA) and that about surgical resection without NAC due to several medical reasons (SMR) to RFS. The patients with SA were significantly lower-risk group for recurrence than those with SMR, and the 5-year RFS rates of the group of SA and that of SMR were 66.0 and 27.7% respectively (*p* < 0.001) (Fig. 5a). The combination of the TMTV and these groups significantly stratified high-risk for recurrence as following order: TMTV < 3.82 SA and SMR (5-yr RFS, 77.9%), TMTV ≥3.82 SA (50.0%), and TMTV ≥3.82 SMR (20.4%) respectively (Fig. 5b), and the characteristics of these groups are shown in Table 8. The patients with TMTV < 3.82 include both SA and SMR groups because the number of patients with TMTV < 3.82 SMR was only six, and these patients had no recurrence and death during follow-up period. This group had 70 patients (92.1%) with cStage I, 57 patients (75.0%) with pStage I, and a few patients with PET-N-positive and with pathological LNMs ≥3. Another two groups had more patients with pathological LNMs ≥3 and with advanced pStage III-IV than those with TMTV < 3.82 SA and SMR. TMTV ≥3.82 SMR group had the highest number of PET-N-positive and pathological LNMs ≥3 patients than any other group.

**Table 8:** The characteristic of thoracic ESCC patients with combined TMTV and the group of SA and/or SMR

	A	B	C
Total patients	76	55	32
MR: absent/present	71/5	55/0	0/32
PET-N: negative/positive	74/2	55/0	9/23
cStage (I/II/III/IV)	70/5/1/0	30/17/8/0	0/13/18/1
pStage (I/II/III/IV)	57/14/5/0	18/19/15/3	0/8/17/7
Number of pathological LNMs (2</≥3)	75/1	46/9	13/19
Postoperative therapy: absent/present	70/6	38/17	11/21
Recurrence, negative/positive	70/6	31/24	11/21

*ESCC* Esophageal squamous cell carcinoma, *TMTV* Total metabolic tumor volume, *pathological LNMs* Pathological lymph node metastases, *MR* Medical reason

PET-N-positive ^18^F-FDG uptake on PET observed in lymph nodes within a three-field region, including

M1LYM of the supraclavicular, cervical paratracheal and celiac artery lymph nodes

A: TMV < 3.82 SA and SMR B: TMTV≥3.82 SA C: TMTV≥3.82 SMR

*SA* Surgical resection alone, *SMR* Surgical resection without NAC due to several medical reasons

### The failure patterns after surgical resection according to TMTV

We compared the failure patterns based on the cut-off value of TMTV for all patients (Table 9). The total recurrence and metastasis rates were both significantly lower in the patients with a TMTV < 3.82 compared to the patients with a TMTV ≥3.82, as follows: TMTV < 3.82, 7.9% (6/76); TMTV ≥3.82, 51.7% (45/87), *p* < 0.001. The hematogenous metastasis rate and the rate of regional lymph node metastasis were both significantly lower in the patients with a TMTV < 3.82 compared to the patients with a TMTV ≥3.82, as follows. Hematogenous metastasis: TMTV < 3.82, 5.2% (4/76); TMTV ≥3.82, 28.7% (25/87), *p* < 0.001. Regional lymph node metastasis: TMTV < 3.82, 2.6% (2/76); TMTV ≥3.82, 13.8% (12/87), *p =* 0.011. No significant difference in local recurrence, dissemination, or distant lymph node metastases was observed between the low-and high-TMTV cut-off value groups.

**Table 9:** The relationship between TMTV and patterns of failure

Pattern of failure	TMTV < 3.8 (pN0–3) (***n =*** 76)	TMTV ≥ 3.8 (pN0–3) (***n =*** 87)	***p***-value
Local recurrence	0 (0.0%)	3 (3.4%)	0.249
Regional lymph node metastasis	2 (2.6%)	12 (13.8%)	0.011
Hematogenous metastasis	4 (5.2%)	25 (28.7%)	< 0.001
Dissemination	1 (1.3%)	6 (6.9%)	0.123
Distant lymph node metastasis, M1(LYM)	0 (0.0%)	5 (5.7%)	0.061
Total recurrence and metastasis	6 (7.9%)	45 (51.7%)	< 0.001

*TMTV* Total metabolic tumor volume, *LYM* Supraclavicular lymph node metastasis

## Discussion

The relationship between the SUVmax and the prognosis of surgically resected esophageal cancer patients has been investigated. Kato et al. suggested that the peakSUVmax may be predictive of patients’ overall survival (OS); the 5-year OS rate for patients with a peak SUV ≥4.5 was 47% in their series, and the 5-year OS rate for those with a peakSUV < 4.5 was 76% [19]. However, Omloo et al. proposed that the SUVmax was not an independent prognostic factor for disease-free survival (DFS) [20]. The usefulness of the SUVmax for predicting the outcomes of resected esophageal cancer patients is thus controversial.

Investigations of MTV in esophageal cancer patients undergoing curative surgical resection alone have also been reported. A study by Hyun et al. showed that the MTV of the primary tumor was a better independent prognostic factor for OS than the SUVmax in 151 esophageal cancer patients [17]. Shum et al. suggested that the pretreatment MTV of the primary tumor was a novel marker for the OS of 26 patients with ESCC treated with curative surgery [21].

There have been only a few investigations of the TMTV and wTLG for predicting the prognosis of ESCC patients who have undergone only a curative surgical resection. Park et al. reported that rather than the SUVmax and MTV, the wTLG was the only significant prognostic factor for both the DFS and OS of 50 ESCC patients [22]. In their study of 103 esophageal cancer patients, Foley et al. indicated that the TLG of the primary tumor, the metastatic length of disease, and the PET/CT total local nodal metastasis count were more important prognostic indicators for the patients’ survival than the TMTV [23]. Our present findings differ from these past reports. In most of the previous investigations, the correlation between the ^18^F-FDG uptake in the primary tumor and the prognosis of esophageal cancer patients was evaluated; the previous investigations included both ESCC patients and non-SCC patients; and the number of patients in the past investigations is lower than that of the present study.

Our study revealed that TMTV demonstrated consistently better performance than any other PET parameters in predicting recurrence until 72 months in a time-dependent ROC analysis, and the multivariate analysis revealed that TMTV was a more significant prognostic factor for RFS than other PET parameters in both models. The total recurrence and metastasis rates were both significantly higher in the patients with a TMTV ≥3.8; in particular, the occurrence rates of hematogenous metastasis and regional lymph node metastasis were significantly related to that TMTV value. Our results demonstrated that the TMTV was an independent prognostic factor for postoperative recurrence in resectable thoracic ESCC patients, and we thus propose that TMTV could be a useful preoperative risk factor for predicting postoperative recurrence in this patient population.

In our thoracic ESCC patient series, the N stage was a significant prognostic factor for predicting postoperative recurrence. The N stage has been classified according to the number of regional lymph node metastases instead of the location of lymph metastases since the 7th edition of TNM staging [13]. This N staging system is made based on survival with a significant difference between the survival curves of N0–N1 and N2–N3 [24]. Our data are agreement with this.

In the present study, the use of the TMTV enabled us to stratify the patients at high risk for recurrence among ESCC patients with cN0–1, pN0–1, and SA and SMR. Our analyses revealed that the patients with a TMTV < 3.8 cN0–1, TMTV< 3.8 pN0, TMTV < 3.8 pN1, and TMTV < 3.8 SA and SMR were at relatively low risk for recurrence. Conversely, the patients with a TMTV ≥3.8 cN0, TMTV ≥3.8 cN1, TMTV ≥3.8 pN0, TMTV ≥3.8 pN1, TMTV ≥3.8 SA and TMTV ≥3.8 SMR were at high risk for recurrence.

These data suggest that thoracic ESCC patients with a TMTV < 3.8 might be followed up without NAC or postoperative adjuvant therapy. Conversely, the patients with a TMTV ≥3.8 were at high risk for recurrence, and these patients may require postoperative adjuvant therapy or NAC. In addition, the TMTV ≥3.8 group included quite a few cStage I and pStage I thoracic ESCC patients. Thoracic ESCC patients with both TMTV ≥3.8 and stage I may need to undergo NAC or postoperative adjuvant chemotherapy.

The TMTV may thus be a crucial biomarker for stratifying thoracic ESCC patients at low and high risk for postoperative recurrence. To the best of our knowledge, the data that we obtained in this study have not been reported before, and we propose that the TMTV may be clinically useful for decisions about thoracic ESCC patient management.

The SULpeak was recently reported to be useful for predicting the tumor response and prognosis based on the PET Response Criteria in Solid Tumors (PERCIST) [25]. The SUL reduces dependence on the patients’ weight compared to the standard body weight- normalized SUV, and the SULpeak reduces the potential inconsistency of single-pixel measurements due to noise. The hSULpeak was not shown to be an independent prognostic factor for RFS following curative surgical resection in the present study, but the SULpeak has been reported to be useful for discriminating pathological responders and non-responders to NAC among ESCC patients [26]. The number of clinical investigations of SULpeak values in malignant tumor patients is less than that of other PET parameters, and the clinical usefulness of the SULpeak for malignant tumors remains under discussion.

This study has some limitations. It is retrospective study of thoracic ESCC patients surgically treated some time ago. Currently the eighth edition AJCC/UICC staging is used. However, we used the seventh edition AJCC/UICC staging in this study because the management of these thoracic ESCC patients was determined based on this edition. An investigation of ^18^F-FDG-PET/CT for predicting prognosis using the eighth edition of AJCC/UICC staging in thoracic ESCC patients should be conducted in a future study.

In addition, there is a possibility of patient selection bias. Thoracic ESCC patients who underwent NAC or NACRT were excluded from this study so that we could investigate the direct relationship between postoperative recurrence and PET parameters. Although NAC plus surgery is the currently recommended standard therapy for cStage II/III ESCC patients, surgery alone or surgery plus postoperative chemotherapy is performed for patients who are practically unable to ingest food due to stenosis or because of any factor that interferes with chemotherapy in the clinical setting [27]. In the present study, NAC was not administered to 63 patients with cStage II/III/IV due to several medical reasons, which led to heterogeneity of the patient population. We used ^18^F-FDG PET/CT to determine eligible patients for NAC for thoracic ESCC. Yasuda et al. investigated the usefulness of a lymph node evaluation by initial ^18^F-FDG PET in prediction of postoperative recurrence for resectable ESCC patients without induction therapy [8]. They suggested that resectable thoracic ESCC patients with PET-N-positive were likely to exhibit ≥3 pathological LNMs and a higher rate of postoperative distant recurrences, resulting in a much lower 5-year RFS compared with those with PET-N-negative (29.5 vs 75.1%, respectively). [8.28]. Aggressive NAC was done to thoracic ESCC patients with PET-N-positive to suppress postoperative recurrence, reducing the number of pathological LNMs, and improving 5-year RFS twofold [12, 28]. In another investigation, they demonstrated that PET-N-negative status predicts ≤2 pathological LNMs and longer 5-year RFS compared with PET-N-positive in resectable thoracic ESCC patients after NAC (69.0 vs 20.0%) [28]. Thus, PET-N status is an important NAC treatment criterion for evaluating prognosis and deciding the patient management, and this criterion has been used before in clinical setting. PET-N-positive means candidate for NAC, and we carried out NAC followed by surgical resection for these patients Alternatively, thoracic ESCC patients with PET-N-negative underwent curative surgical resection without NAC. The difference in background of cStage II-IV patients between patients who underwent NAC and those who did not mainly depend on PET-N status.

Although surgery is conducted under the diagnosis of cStage I, there may be cases in which the disease stage is found to be pStage II/III after surgical resection, and postoperative adjuvant chemotherapy is recommended in such cases [27]. Postoperative adjuvant chemotherapy with cisplatin and fluorouracil has been reported to be better able to prevent relapse in patients with esophageal cancer than surgery alone, but unfortunately there is no clear evidence that postoperative chemotherapy improves the OS of patients undergoing curative resection [27, 29]. If the optimal regimen for postoperative chemotherapy is clarified, TMTV may be a useful index for selecting the patients who should undergo postoperative therapy.

We included the patients with a TMTV< 3.8 cN1 or TMTV< 3.8 SMR as TMTV< 3.8 cN0–1or TMTV< 3.8 SA and SMR respectively. In this study, the number of patients with TMTV< 3.8 cN1 and TMTV< 3.8 SMR was low. These investigations for predicting prognosis should be done in a greater number of ESCC patients. A standard threshold for delineating ^18^F-FDG uptake for the MTV has not been established. Volumetric parameters are affected by the delineation method and threshold. The relative threshold could result in an overestimation of the MTV in a tumor with low metabolic activity, whereas a fixed value (e.g., SUV 2.5) could lead to underestimation [30]. We used volumetric parameters obtained from a threshold of 40% of the maximum peak activity because the threshold value of the SUVmax is the most commonly used in clinical settings. Our results should be confirmed by similar studies performed prospectively at different institutions.

## Conclusions

The results of our analyses demonstrated that the TMTV was a more significant independent prognostic factor for RFS than any other PET parameters in resectable thoracic ESCC patients. The TMTV may be a more promising biomarker for the identification of patients at high risk for postoperative recurrence, and it could be useful for deciding the patient management in thoracic ESCC patients.

## Supplementary Information

**Additional file 1.**

**Additional file 2.**

**Additional file 3.**

**Additional file 4.**

## Data Availability

The datasets used and /or analyzed during the current study available from the corresponding author on reasonable request.

## Abbreviations

ESCC: Esophageal squamous cell carcinoma
^18^F-FDG-PET/CT: Fluorine-18 fluorodeoxyglucose positron emission tomography/computed tomography
RFS: Relapse free survival
HR: Hazard ratio
95% CI: 95% confidential interval
LNMs: Lymph node metastases
NACRT: Neoadjuvant chemoradiotherapy
NAC: Neoadjuvant chemotherapy
TNM: The tumor-node metastasis
peakSUVmax: Peak maximum standardized uptake value
TMTV: Total metabolic tumor volume
CRT: Chemoradiotherapy
wTLG: Whole-body total lesion glycolysis
hSULpeak: Highest lean body mass peak standardized uptake value
PET-N-positive: ^18^F-FDG uptake on PET observed in lymph nodes within a three-field region, including M1LYM of the supraclavicular, cervical paratracheal and celiac artery lymph nodes
VOIs: Volumes of interest
ROI: Region of interest
ROC: Receiver operating characteristics curve
AUC: The area under the curve
SA: Surgical resection alone
SMR: Surgical resection without NAC due to several medical reasons
LYM: Supraclavicular lymph node metastasis
