# Supplementary material for: The usefulness of the total metabolic tumor volume for predicting the postoperative recurrence of thoracic esophageal squamous cell carcinoma
Source: BMC Cancer. 2022 Nov 15;22:1176. doi: 10.1186/s12885-022-10281-4 (PMC9664655; doi:10.1186/s12885-022-10281-4)
Supplement: Supplementary file 2 — Additional file 2. [file 12885_2022_10281_MOESM2_ESM.docx]

| **Suppl. Table S1**. Reasons for curative surgical resection without NAC in cStage II-IV patients | |
| --- | --- |
| Characteristics | (n=63) |
| PET-N-negative alone | 26 |
| PET-N negative and medical reasons | 12 |
| medical reasons |  |
| Renal dysfunction | 3 |
| Liver dysfunction (alcoholic liver disease or chronic hepatitis B or C type) | 3 |
| Both renal and liver dysfunction | 1 |
| Both renal and cardiac dysfunction | 1 |
| Cardiac dysfunction due to coronary heart disease | 1 |
| Difficulty of long-term cancellation of imatinib for chronic myeloid leukemia | 1 |
| Difficulty in oral ingestion due to stenosis | 1 |
| Past history of CRT for primary lung cancer and surgical resection for hypopharyngeal and oral cancers | 1 |
| PET-N-positive | 25 |
| medical reasons |  |
| Diagnosis as local disease due to lymph node metastasis adjacent to primary tumor | 7 |
| Multiple cerebral infarctions and advanced age | 3 |
| Liver dysfunction (alcoholic liver disease or chronic hepatitis B or C type) | 3 |
| No agreement to NAC from patients | 3 |
| Advanced age | 2 |
| After effect of cerebral hemorrhage | 1 |
| Multiple cerebral infarctions, diabetes mellitus and hypertension | 1 |
| Coronary heart disease and synchronous early gastric cancer | 1 |
| Renal dysfunction | 1 |
| Chronic hepatitis and myocardial infarction | 1 |
| Past history of radiotherapy for tongue cancer recurrence | 1 |
| Synchronous malignant lymphoma | 1 |
| PET-N-positive: ^18^F-FDG uptake on PET observed in lymph nodes within a three-field region, including M1LYM of the supraclavicular, cervical paratracheal and celiac artery lymph nodes, CRT: chemoradiotherapy, NAC: neoadjuvant chemotherapy, PET-N-negative and medical reasons and PET-N-positive: These cases were considered as candidate for NAC, unfortunately it was not performed because of several medical reasons. Diagnosis as local disease due to lymph node metastasis adjacent to primary tumor: These patients underwent curative surgical resection without NAC as local disease. | |
