# Supplementary material for: The usefulness of the total metabolic tumor volume for predicting the postoperative recurrence of thoracic esophageal squamous cell carcinoma
Source: BMC Cancer. 2022 Nov 15;22:1176. doi: 10.1186/s12885-022-10281-4 (PMC9664655; doi:10.1186/s12885-022-10281-4)
Supplement: Supplementary file 3 — Additional file 3. [file 12885_2022_10281_MOESM3_ESM.docx]

| **Suppl. Table S2.** The ^18^F-FDG uptake and clinicopathological factors in thoracic ESCC patients | | | | | | |
| --- | --- | --- | --- | --- | --- | --- |
|  | **peakSUVmax**  **<4.43** | **peakSUVmax**  **≥4.43** | **p-value** | **hSULpeak**  **<2.64** | **hSULpeak**  **≥2.64** | **p-value** |
|  | **(n=71)** | **(n=92)** |  | **(n=68)** | **(n=95)** |  |
| Age: |  |  |  |  |  |  |
| <66 | 36 | 42 | 0.522 | 36 | 42 | 0.340 |
| ≥ 66 | 35 | 50 |  | 32 | 53 |  |
| Gender: |  |  |  |  |  |  |
| Male | 62 | 73 | 0.181 | 58 | 77 | 0.454 |
| Female | 9 | 19 |  | 10 | 18 |  |
| Location: |  |  |  |  |  |  |
| Upper-Middle | 54 | 67 | 0.64 | 52 | 69 | 0.581 |
| Lower | 17 | 25 |  | 16 | 26 |  |
| Upper | 7 | 18 | 0.088 | 8 | 17 | 0.284 |
| Middle-Lower | 64 | 74 |  | 60 | 78 |  |
| Clinical factors: |  |  |  |  |  |  |
| cTstage: |  |  |  |  |  |  |
| cT1,T2 stage | 71 | 57 | <0.001 | 68 | 60 | <0.001 |
| cT3,T4 stage | 0 | 35 |  | 0 | 35 |  |
| cN stage: |  |  |  |  |  |  |
| cN0 stage | 68 | 45 | <0.001 | 65 | 48 | <0.001 |
| cN1, N2 stage | 3 | 47 |  | 3 | 47 |  |
| cM stage: |  |  |  |  |  |  |
| cM0 stage | 71 | 91 | 1.00 | 68 | 94 | 1.00 |
| cM1 (LYM) stage | 0 | 1 |  | 0 | 1 |  |
| cStage: |  |  |  |  |  |  |
| cStage I, II | 71 | 64 | <0.001 | 68 | 67 | <0.001 |
| cStage III, IV | 0 | 28 |  | 0 | 28 |  |
| Pathological factors: |  |  |  |  |  |  |
| pTstage |  |  |  |  |  |  |
| pT1,T2 stage | 71 | 43 | <0.001 | 68 | 46 | <0.001 |
| pT3,T4 stage | 0 | 49 |  | 0 | 49 |  |
| pN stage: |  |  |  |  |  |  |
| pN0, N1 stage | 67 | 66 | <0.001 | 64 | 69 | <0.001 |
| pN2, N3 stage | 4 | 26 |  | 4 | 26 |  |
| pM stage: |  |  |  |  |  |  |
| pM0 stage | 71 | 88 | 0.133 | 68 | 91 | 0.141 |
| pM1 (LYM) stage | 0 | 4 |  | 0 | 4 |  |
| pStage: |  |  |  |  |  |  |
| pStage I, II | 67 | 49 | <0.001 | 64 | 52 | <0.001 |
| pStage III, IV | 4 | 43 |  | 4 | 43 |  |
| No. of PET-N-positive |  |  |  |  |  |  |
| 0 | 69 | 69 | <0.001 | 66 | 72 | <0.001 |
| 1 | 2 | 19 |  | 2 | 19 |  |
| 2 | 0 | 4 |  | 0 | 4 |  |
| Events: |  |  |  |  |  |  |
| No recurrence | 63 | 49 | <0.001 | 61 | 51 | <0.001 |
| Recurrence | 8 | 43 |  | 7 | 44 |  |
| ^18^F-FDG fluorine-18 fluorodeoxyglucose, hSULpeak: highest lean body mass, peak standardized uptake value, LYM: supraclavicular lymph node metastasis, peakSUVmax: peak maximum standardized uptake value, ESCC: esophageal squamous cell carcinoma, PET-N-positive: ^18^F-FDG uptake on PET observed in lymph nodes within a three-field region, including M1LYM of the supraclavicular, cervical paratracheal and celiac artery lymph nodes. | | | | | | |
