# Supplementary material for: The usefulness of the total metabolic tumor volume for predicting the postoperative recurrence of thoracic esophageal squamous cell carcinoma
Source: BMC Cancer. 2022 Nov 15;22:1176. doi: 10.1186/s12885-022-10281-4 (PMC9664655; doi:10.1186/s12885-022-10281-4)
Supplement: Supplementary file 4 — Additional file 4. [file 12885_2022_10281_MOESM4_ESM.docx]

| **Suppl. Table S3.** The ^18^F-FDG uptake and clinicopathological factors in the thoracic ESCC patients | | | | | | |
| --- | --- | --- | --- | --- | --- | --- |
|  | **TMTV <3.82 (n=76)** | **TMTV ≥3.82**  **(n=87)** | **p-value** | **wTLG <13.46**  **(n=78)** | **wTLG ≥13.46**  **(n=85)** | **p-value** |
| Age: |  |  |  |  |  |  |
| <66 | 37 | 41 | 0.843 | 40 | 38 | 0.401 |
| ≥66 | 39 | 46 |  | 38 | 47 |  |
| Gender: |  |  |  |  |  |  |
| Male | 67 | 68 | 0.091 | 68 | 67 | 0.158 |
| Female | 9 | 19 |  | 10 | 18 |  |
| Location: |  |  |  |  |  |  |
| Upper- Middle | 60 | 61 | 0.198 | 60 | 61 | 0.452 |
| Lower | 16 | 26 |  | 18 | 24 |  |
| Upper | 12 | 13 | 0.881 | 11 | 14 | 0.675 |
| Middle-Lower | 64 | 74 |  | 67 | 71 |  |
| Clinical factors: |  |  |  |  |  |  |
| cTstage: |  |  |  |  |  |  |
| cT1,T2 stage | 75 | 53 | <0.001 | 77 | 51 | <0.001 |
| cT3,T4 stage | 1 | 34 |  | 1 | 34 |  |
| cN stage: |  |  |  |  |  |  |
| cN0 stage | 71 | 42 | <0.001 | 74 | 39 | <0.001 |
| cN1,N2 stage | 5 | 45 |  | 4 | 46 |  |
| cM stage: |  |  |  |  |  |  |
| cM0 stage | 76 | 86 | 1.000 | 78 | 84 | 1.000 |
| cM1 (LYM) stage | 0 | 1 |  | 0 | 1 |  |
| cStage: |  |  |  |  |  |  |
| cStage I, II | 76 | 60 | <0.001 | 78 | 57 | <0.001 |
| cStage III, IV | 0 | 27 |  | 0 | 28 |  |
| Pathological factors: |  |  |  |  |  |  |
| pTstage: |  |  |  |  |  |  |
| pT1,T2 stage | 72 | 42 | <0.001 | 77 | 37 | <0.001 |
| pT3,T4 stage | 4 | 45 |  | 1 | 48 |  |
| pN stage: |  |  |  |  |  |  |
| pN0, N1 stage | 74 | 59 | <0.001 | 73 | 60 | <0.001 |
| pN2, N3 stage | 2 | 28 |  | 5 | 25 |  |
| pM stage: |  |  |  |  |  |  |
| pM0 stage | 75 | 84 | 0.623 | 77 | 82 | 0.622 |
| pM1 (LYM) stage | 1 | 3 |  | 1 | 3 |  |
| pStage: |  |  |  |  |  |  |
| pStage I, II | 71 | 45 | <0.001 | 72 | 44 | <0.001 |
| pStage III, IV | 5 | 42 |  | 6 | 41 |  |
| No. of PET-N-positive |  |  |  |  |  |  |
| 0 | 74 | 64 | <0.001 | 75 | 63 | <0.001 |
| 1 | 2 | 19 |  | 3 | 18 |  |
| 2 | 0 | 4 |  | 0 | 4 |  |
| Events: |  |  |  |  |  |  |
| No recurrence | 70 | 42 | <0.001 | 70 | 42 | <0.001 |
| Recurrence | 6 | 45 |  | 8 | 43 |  |
| ESCC: esophageal squamous cell carcinoma, TMTV: total metabolic tumor volume, wTLG: whole-body total lesion glycolysis, LYM: supraclavicular lymph node metastasis, ^18^F-FDG: fluorine-18 fluorodeoxyglucose, PET-N-positive: ^18^F-FDG uptake on PET observed in lymph nodes within a three-field region, including M1LYN of the supraclavicular, cervical paratracheal and celiac artery lymph nodes. | | | | | | |

.
